# The Clinical Significance of Lymph Node Ratio and Ki-67 Expression in Papillary Thyroid Cancer

**DOI:** 10.1007/s00268-021-06070-y

**Published:** 2021-04-07

**Authors:** Helene Lindfors, Catharina Ihre Lundgren, Jan Zedenius, C. Christofer Juhlin, Ivan Shabo

**Affiliations:** 1 Department of Surgery, Sundsvall Hospital, 851 86 Sundsvall, Sweden; 2 Department of Molecular Medicine and Surgery, Karolinska Institutet, 171 77 Stockholm, SE Sweden; 3 Department of Breast, Endocrine Tumors and Sarcoma, Karolinska University Hospital, 171 76 Stockholm, SE Sweden; 4 Department of Oncology-Pathology, Karolinska Institutet, 171 77 Stockholm, SE Sweden; 5 Department of Pathology and Cytology, Karolinska University Hospital, 171 76 Stockholm, SE Sweden

## Abstract

**Background:**

The N stage in papillary thyroid cancer (PTC) is an important prognostic factor based on anatomical localization of cervical lymph nodes (LNs) only and not the extent of lymphatic metastasis. In this retrospective study, the clinical significance of lymph node ratio (LNR) and tumor cell proliferation in relation to the conventional classification of PTC was explored.

**Methods:**

Patients diagnosed with PTC at the Karolinska University Hospital in Stockholm, Sweden, during the years 2009–2011 were included. The LNR, defined as the number of metastatic LNs divided by the total number of LNs investigated, and the Ki-67 index were analyzed in relation to clinical data.

**Results:**

The median number of LN removed was 16 with the following N stage distribution: N0 (26%), N1a (45%), and N1b (29%). A Ki-67 index of ≥3% was significantly correlated with the presence of metastases and tumor recurrence with a sensitivity of 50% and specificity of 80% (*p* = 0.015). Lymph node ratio ≥21% was related to tumor recurrence with sensitivity of 89% and specificity of 70% (*p* = 0.006). Patients with LN metastases in the lateral cervical compartment only had significantly lower LNR (14.5%) compared to those with both central and lateral cervical metastases (39.5%) (*p* = 0.004) and exhibited no tumor recurrence. Increased Ki-67 index was significantly related to LNR ≥21% (*p* = 0.023) but was not associated with N stage.

**Conclusions:**

The Ki-67 proliferation index and LNR may better reflect the malignant behavior of PTC compared to the anatomical classification of LN metastases solely.

## Introduction

Papillary thyroid carcinoma (PTC) constitutes the most common type of thyroid cancer and exhibits excellent prognosis with a 10-year survival rate of 90–95%. However, 10–20% of cases with PTC demonstrate more aggressive behavior with early tumor recurrences. At the time of diagnosis, locoregional cervical lymph node (LN) metastases are found in 20–90% of patients [1].

The N stage in PTC is based on the anatomical location of the LN metastases, classified into central LN (N1a) or lateral LN (N1b), and has no rational correlation to the biology of PTC [2]. Lymph node ratio (LNR) is the proportion of metastatic LN of the total number of LN diagnosed following surgery. In PTC [3] and other solid tumors, such as bladder cancer [4] and breast cancer [5], LNR is suggested as a prognostic variable, better reflecting the tumor burden and recurrence [6, 7].

The Ki-67 index is a commonly used biomarker for assessing proliferation, constituting a prognostic indicator in several types of tumors such as breast cancer and neuroendocrine neoplasia [8, 9]. The prognostic and predictive value of Ki-67 in assessment of PTC is still controversial. However, several studies show that an increased Ki-67 index in PTC is associated with an increased risk of tumor recurrence and poor survival [10, 11].

In this study, we hypothesize that LNR and tumor cell proliferation better reflect the tumor biology and malignant behavior of PTCs compared to the anatomical classification of LN metastasis only.

## Material and methods

### Patient material

All patients diagnosed for PTC at the Karolinska University Hospital, Stockholm, Sweden, during 2009–2011 were retrospectively included. In case of a preoperatively confirmed cytological diagnosis of PTC, a systematic ultrasound-guided LN mapping of all cervical regions was performed by experienced radiologists to explore the presence of pathologically enlarged LNs. In the presence of pathologically enlarged cervical LNs, these were examined with fine needle aspiration and cytology to confirm the PTC metastasis. All patients were treated with total thyroidectomy and clearance of central cervical LN in accordance with the Swedish national guidelines prevailing the study period, which in turn were based on ATA [12] and ETA [13] recommendations. In cases with preoperatively confirmed LN metastases in the lateral cervical compartments, lateral LN dissection was performed during the primary operation.

About 6–8 weeks after surgery, the patients were offered adjuvant radioiodine irradiation therapy (RAI) and post-irradiation thyroid scintigraphy. The patients had regular follow-up with clinical and biochemical controls. The first control was 9–12 months after completing the primary treatment and post-therapy scintigraphy. In case of recurrence-free outcomes, the subsequent controls then continued annually. All cases with biochemical or clinical signs of tumor recurrence were investigated by ultrasound examination of the neck, and all pathologically enlarged LN were examined by fine needle aspiration cytology. Recurrence of PTC was defined as the first biochemical recurrence, clinical and/or cytologically confirmed in locoregional LN or distant metastasis.

The main hypothesis of the study was that the tumor biology of PTC, exemplified by Ki-67 expression, might relate to the presence of LN metastasis and metastatic burden, estimated as LNR. The primary endpoints of the study were the presence of LN metastases and tumor recurrence explored in relation to Ki-67 index, LNR, and clinical data. The secondary endpoints were recurrence-free survival (RFS), examined in relation to Ki-67 index, and LNR as well Ki-67 index in relation to clinical data. We chose RFS as secondary endpoint because it represents a definitive answer that was not expected or feasible to assess as an independent primary endpoint. Moreover, Ki-67 as secondary endpoint is expected to provide additional clinical information, exploring the biological impact of Ki-67 index and clinical significance toward a more personalized treatment concept.

### Immunohistochemistry and histopathologic examination

Histopathological diagnostics were performed according to clinical routine practice by endocrine pathologists at the Department of Pathology, Karolinska University Hospital, Stockholm, Sweden. Given the time of data collection, the PTC was diagnosed using the guidelines of the 2004 WHO classification of endocrine tumors, and the TNM staging was according to the 7th edition of the American Joint Committee on Cancer. The following data were obtained: total number of observed LNs and number of LNs with metastasis in all cervical regions, number and size of the primary PTCs, presence of extrathyroidal extension, presence of thyroiditis, and the status of the resection margins.

Immunostaining of all primary tumors and LN metastases was performed according to clinical laboratory standards. Micrometastases are defined as clusters of tumor cells that are <2.0 mm in diameter [14]. At our pathology department, primary thyroid tumors and the largest LN metastasis are routinely assessed for Ki-67 immunohistochemistry. The Ki-67 labeling index is calculated by manual counting of the amount of Ki-67 positive cells (only nuclear staining) divided by the total amount of tumor cells in “hot-spot” regions, counting at least 2000 cells. The LNR was defined as the total number of metastatic LNs divided by the total number of LNs retrieved from the central and lateral cervical compartment.

### Statistical analysis

Statistical analyses were performed using SPSS statistics software, version 26 (IBM Inc., Chicago, USA). Pearson’s Chi-square test was applied to evaluate the relationship between tumor recurrence and the presence of metastasis in relation to clinical data such as tumor size, gender, and tumor stadium. To evaluate the performance of means for LNR and Ki-67 expression rates as cutoff values, we used receiver operating characteristic (ROC) analysis. The sensitivity and 1-minus specificity data over tumor recurrence-free survival as outcomes were used. Area under curve (AUC) was calculated with 95% CIs for LNR and rates of Ki-67 expression means as a dichotomous variable.

Comparison of clinical data, LNR and proportion of cancer cells expressing Ki-67 was achieved by one-way analysis of variance (ANOVA) together with a post hoc Bonferroni’s test. Survival rates were estimated according to Kaplan–Meier curves and were based on recurrence-free survival (RFS) with median follow-up of 77 months (range of 2–100 months). The statistical significance of differences between survival rates was determined by the log-rank test. *P* < 0.05 was considered statistically significant for all measurements.

## Results

Out of 165 patients, 131 (79%) were female. The patients had a median age of 47 years (range 12–83). In 64 (38.8%) patients, the T stage was ≥T3. The primary tumors were multifocal in 61 (37%) and radically removed in 146 (89%) patients. Extrathyroidal extension was found in 52 (32%) patients (Table 1). At the time of primary surgery, LN metastases were found in 74 (66%) patients.

**Table 1:** Patient characteristics

	Patient N (%)
*Gender*	
Male	34 (21)
Female	131 (79)
*Age-group*	
<40 years	60 (36)
40–49 years	41 (25)
50–59 years	24 (14)
60–69 years	19 (12)
≥70 years	21 (13)
*T-stage*	
pTx	1 (0.6)
pT1a	47 (28.5)
pT1b	29 (17.6)
pT2	24 (14.5)
≥pT3	64 (38.8)
*N-stage*	
N0	26 (16)
N1a	45 (27)
N1b	29 (18)
Nx	65 (39)
*Lymph node ratio*	
<21%	54 (54)
≥21%	46 (46)
Nx	65*
*Thyroiditis*	
No	125(76)
Yes	40 (24)
*Multifocal*	
Unifocal	103 (63)
Multifocal	61 (37)
T0 (missing data)	1*
*Radical*	
No	19 (11)
Yes	146 (89)
*Ki-67 index*	
≤1%	31 (24)
1–2%	38 (29)
2–3%	42 (32)
>3%	20 (15)
Missing data	34*
*Extra-glandular extension*	
No	113 (68)
Yes	52 (32)
*Type of metastases*	
Micro	12 (16)
Macro	62 (84)

### Tumor recurrence in relation to the Ki-67 index and LNR

Out of 165 patients, 65 (39%) were excluded from further LNR analysis as they had less than 6 LNs (Nx stage) removed during primary surgery. Three patients with Nx stage later exhibited tumor recurrences, of which one patient never underwent LN dissection as thyroidectomy was performed due to goiter; in the second patient, one tumor-free central LN was removed. The third patient already had brain metastases and underwent thyroidectomy in order to receive RAI therapy.

**Fig. 1:**
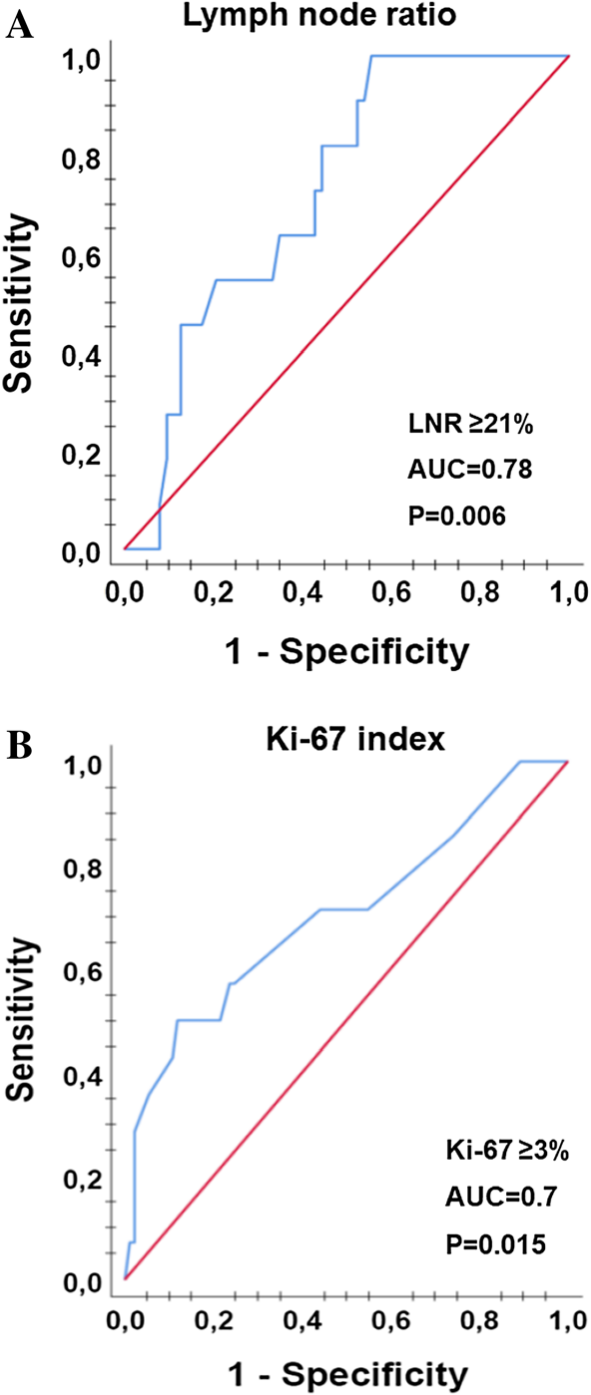
A receiver operating characteristic (ROC) curve illustrating the ability of lymph node ratio (LNR) and the Ki-67 expression by cancer cells in predicting tumor recurrence of papillary thyroid cancer. **a** Tumors with LNR rates ≥ 21% predicted PTC relapse with a sensitivity of 89%, specificity of 79%, and area under cover (AUC) of 0.78 (*p* = 0.006). **b** Ki-67 index of ≥3% predicted PTC recurrence with a sensitivity of 50%, specificity of 80%, and AUC of 0.70 (*p* = 0.015)

In the remaining 100 patients, the median number of total LNs removed per patient was 16 (range 6–81). The N stage distribution was N0 in 26 (26%), N1a in 45 (45%), and N1b in 29 (29%) patients (Table 1). Based on ROC analysis and in relation to tumor recurrence, the highest AUC identified for LNR ≥ 21% was 0.78 with a sensitivity of 89% and specificity of 70% (*p* = 0.006). For Ki-67, a labeling index of ≥3% was predictive for tumor recurrences with a sensitivity of 50%, specificity of 80%, and AUC of 0.7 (*p* = 0.015) (Fig. 1).

### Ki-67 index

**Fig. 2:**
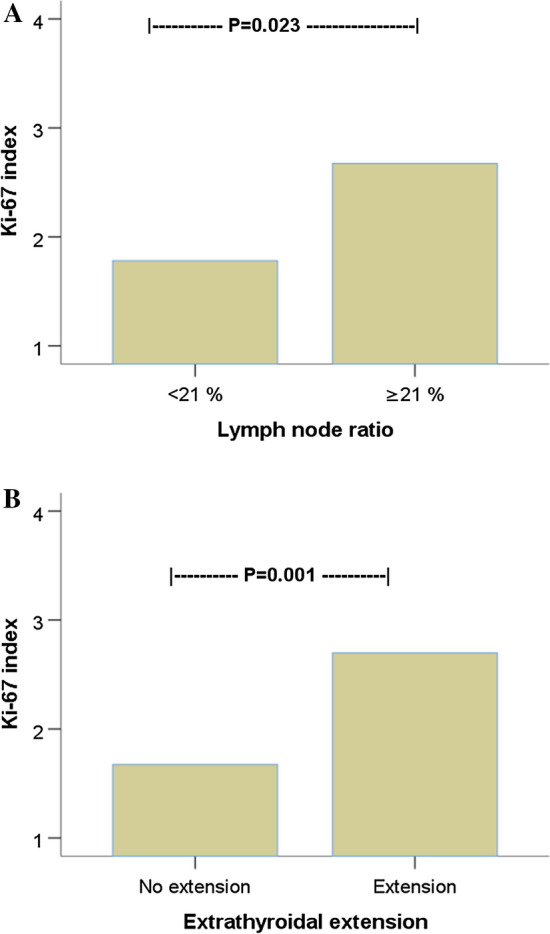
Analysis of variance (*ANOVA*) comparing the means of Ki-67 index in papillary thyroid cancer in relation to **a** lymph node ratio (LNR) with cutoff value of 21% and **b** extrathyroidal tumor extension

In patients with LNR ≥ 21%, the mean Ki-67 index in primary tumors was 2.7%, which was significantly higher than in patients (mean Ki-67 index 1.8%) with LNR < 21% (*p* = 0.023). Tumors with extrathyroidal extension exhibited a significantly increased Ki-67 index (2.7%) compared to those without extrathyroidal extension (1.7%) (*p* = 0.001) (Fig. 2). No statistically significant difference in Ki-67 index was found in relation to age, the presence of thyroiditis, T stage, or N stage.

### The presence of lymph node metastases

At the time of surgery, LN metastases were inversely related to age, a difference in age distribution that could not be found in patients without a LN metastasis. Of all patients with LN metastases, 44 (60%) had tumors with T stage ≥3. Within the patient group with ≥pT3 tumors, 79% (44 out of 56) (*p* = 0.04) had LN metastasis (Table 2).

**Table 2:** Univariate analysis comparing the presence of cervical lymph node metastasis at the time of diagnosis in relation to clinicopathological data of papillary thyroid cancer

	Lymph node metastasis		P
	No *N* (%)	Yes *N* (%)	
*Gender*			
Female	30 (79)	54 (73)	
Male	8 (21)	20 (27)	0.5
*Age-group*			
<40 years	14 (37)	31 (42)	
40–49 years	8 (21)	20 (27)	
50–59 years	2 (5)	11 (15)	
60–69 years	9 (24)	4 (5)	
≥70 years	5 (13)	8 (11)	0.04
*T-stage*			
pTx	0 (0)	1 (1)	
pT1a	4 (11)	11 (15)	
pT1b	13 (34)	9 (12)	
pT2	9 (24)	9 (12)	
≥pT3	12 (32)	44 (60)	0.01
*Thyroiditis*			
No	28 (74)	54 (73)	
Yes	10 (26)	20 (27)	0.9
Multifocal			
Unifocal	30 (79)	36 (49)	
Multifocal	8 (21)	38 (51)	0.003
*Ki-67 index*			
<3%	29 (85)	43 (66)	
≥3%	5 (15)	22 (34)	0.04
*Extrathyroidal extension*			
No	30 (79)	36 (49)	
Yes	8 (21)	39 (51)	0.002

Lymph node metastases were significantly more frequent in patients with multifocal tumors, Ki-67 index of ≥3%, and extrathyroidal extension. No significant correlation was found between LN metastasis, gender, or the presence of thyroiditis (Table 2).

Micrometastases were found in 12 (16%) of all 74 patients with pathological LN and occurred mainly in patients (10 out of 12, 83%) with T1-2 tumors. Macrometastases were more common in patients (42 out of 62, 68%) with ≥T3 tumors (*p* < 0.001) and were associated with extrathyroidal extension (37 out of 62 patients, 60%). Inversely, micrometastases were found in 1 out of 12 patients (2.6%) with ≥T3 tumors (*p* = 0.001). Macrometastases were significantly related to tumors with Ki-67 index ≥ 3%. Micrometastases were found in 11 out of 12 patients (92%) who had tumors with Ki-67 index < 3%. The corresponding rate in patients with macrometastases was 32 out of 62 patients (39%) (*p* = 0.039). Tumor recurrence was found in patients with macrometastases only.

### Tumor recurrence, Ki-67, LNR, and clinical data

Tumor recurrence was significantly related to advanced tumor stages (≥pT3) (*p* = 0.006). None of the patients with pTx or pT1 tumors exhibited a tumor recurrence (Table 3). The tumor relapsed significantly more often in patients with N1b stage compared to N0 and N1a stages. Comparing within each N stage category, tumor recurrence was significantly more common in patients with N1b stage (10 out of 12, 83%) compared to patients with N1a and N0 stages (1 out of 12, 8.3% each stage) (*p* = 0.01) (Table 3).

**Table 3:** Univariate analysis comparing the recurrence of papillary thyroid cancer in relation to clinicopathological data

	Recurrence of papillary thyroid cancer		P
	No N (%)	Yes N (%)	
*Gender*			
Female	121 (81)	10 (67)	
Male	29 (19)	5 (33)	0.2
*Age-group*			
<40 years	51 (34)	9 (60)	
40–49 years	38 (25)	3 (20)	
50–59 years	24 (16)	0 (0)	
60–69 years	18 (12)	1 (7)	
≥70 years	19 (13)	2 (13)	0.2
*T-stage*			
pTx	1 (0.7)	0 (0)	
pT1a	47 (31.3)	0 (0)	
pT1b	29 (19.3)	0 (0)	
pT2	20 (13.3)	4 (27)	
≥pT3	53 (35.3)	11 (73)	0.006
*N-stage*			
N0	25 (28)	1 (8.3)	
N1a	43 (49)	1 (8.3)	
N1b	20 (23)	10 (83.4)	< 0.001
*Lymph node ratio*			
<21%	53 (60)	1 (8)	
≥21%	35 (40)	11 (92)	0.001
*Thyroiditis*			
No	112 (75)	13 (87)	
Yes	38 (25)	2 (13)	0.3
*Multifocal*			
Unifocal	97 (65)	6 (40)	
Multifocal	52 (35)	9 (60)	0.06
*Radical*			
No	15 (10)	4 (27)	
Yes	135 (90)	11 (73)	0.054
*Ki-67 index*			
<3%	94 (81)	8 (53)	
≥3%	22 (19)	7 (47)	0.015
*Extrathyroidal extension*			
No	108 (72)	5 (33)	
Yes	42 (28)	10 (67)	0.002
*Type of metastases*			
Micro	13 (100)	0 (0)	
Macro	51 (82)	11 (18)	0.1

Tumor recurrence increased significantly in patients with LNR ≥ 21 compared to those with LNR < 21% (*p* = 0.001). Tumor recurrence occurred in 8 out of 102 (8%) patients with tumoral Ki-67 expression <3% and in 7 out of 29 (24%) patients with tumoral Ki-67 rates ≥3% (*p* = 0.015). As expected, tumor recurrence was significantly correlated to extrathyroidal extension (*p* = 0.002) but not to gender, age, or the presence of thyroiditis (Table 3).

### LNR and Ki-67 index in relation to RFS

**Fig. 3:**
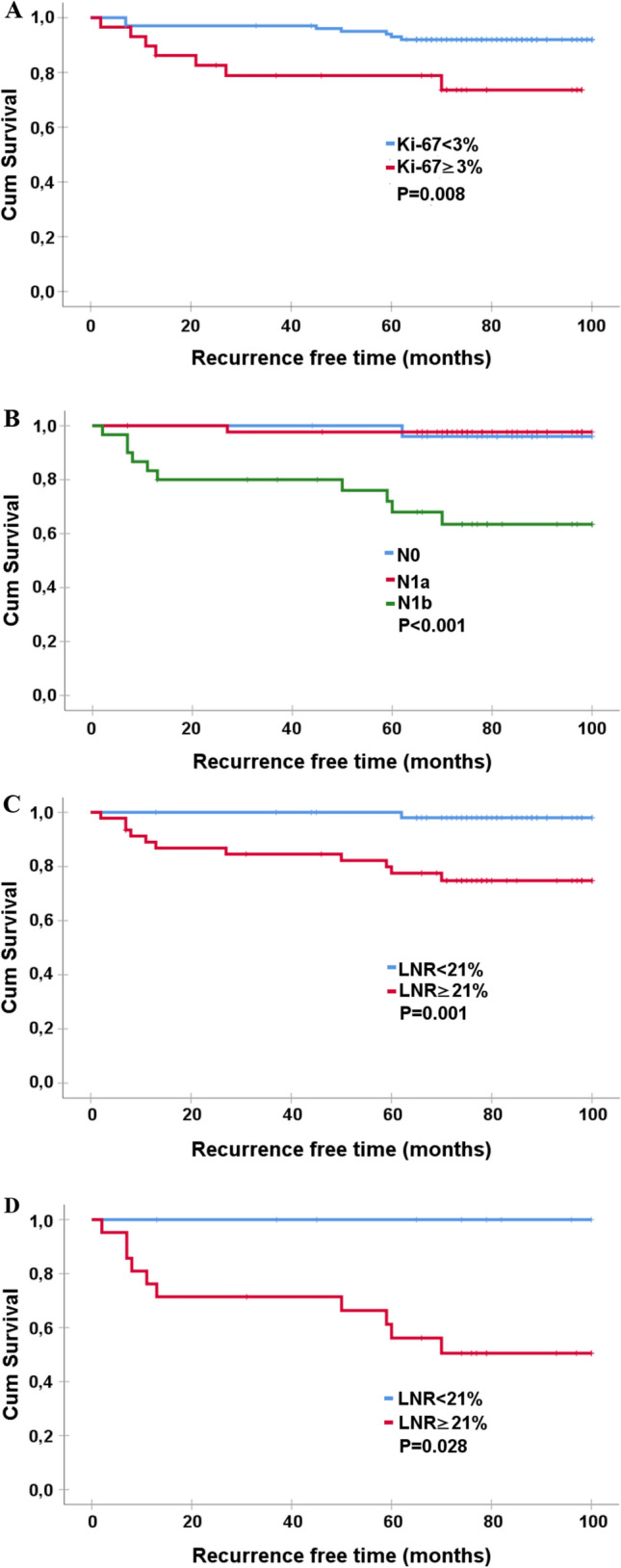
Recurrence-free time in papillary thyroid cancer analyzed as Kaplan–Meier curves comparing recurrence-free survival in relation to **a** Ki-67 index with a cutoff value of 3%, **b** N-stage and **c** lymph node ration with a cutoff value of 21% in patients with papillary thyroid cancer. Panel (D) represents a subgroup survival analysis in patients with N1b tumors in relation to lymph node ratio with a cutoff value of 21%

Patients with tumors displaying a Ki-67 index < 3% had longer RFS (95 months) compared to patients with tumors expressing Ki-67 index ≥ 3% (79 months, *p* = 0.008). Patients with N0 and N1a stage tumors had a similar RFS of 98 months, which was significantly longer than for those with N1b tumors (75 months, *p* < 0.001). The corresponding rates of RFS for patients with LNR < 21% and LNR ≥ 21% were 99 and 82 months, respectively (*p* = 0.001) (Fig. 3a–c).

To explore whether the localization of LN metastasis was significant for RFS, we performed a subgroup analysis in which we classified patients with N1b stage into a group with both central and lateral LN metastases (Na + b group, *n* = 23) and those who only had lateral LN metastases (Nb group, *n* = 7). In Na + b group, 10 (43%) patients had tumor recurrence with mean RFS rate of 28.7 months. Interestingly, tumor recurrence was not found in any patients in the Nb group and RFS was comparable to patients in N0 and N1a stages where one patient in respective stage had tumor recurrence. Moreover, patients in Nb group had significantly lower LNR (14.5%) compared to those in Na + b group (39.5%) (*p* = 0.004). When estimating RFS in patients with N1b in relation to LNR, it appears that those with LNR ≥ 21% had mean RFS of 52.8 months. The corresponding RFS rate for patients with LNR < 21% was 65.7 months (*p* = 0.028) (Fig. 3d).

## Discussion

In many solid tumors, increasing number of metastatic nodes is proportional to tumor burden and associated with increased risk of tumor recurrence and mortality [4, 15, 16]. However, the current N stage classification in PTC is based on anatomical location of LN metastases only, which may underestimate the significance and extent of metastatic burden of the disease.

In this study, LNR ≥ 21% was related to tumor recurrence. In previous studies [17–20], LNR rates associated with tumor recurrence in PTC varied between 40 and 70%, which is higher than the rates found in this study. The estimation of LNR is confounded by the number of LN harvested and is affected by the extent of the surgery performed, anatomical variation between patients, and the variability of the pathological examination [3, 21]. Tentatively, the probability that metastatic nodal disease would be left unresected is higher when a smaller LN number is removed. Consequently, it would be logical to maximize the number of LNs harvested during cervical LN dissection to reduce the risk of local recurrence and probably long-term prognosis of PTC [3, 22].

The number of LN harvested during the primary surgery is important. In several tumor types, such as colon cancer, it is generally accepted that a minimum number or “cutoff” of LN are needed to accurately evaluate the nodal status, preventing inadequate sampling and staging of the disease [14, 23]. The accepted number of LNs detected in PTC has traditionally been 6 nodes. Sugitani et al. demonstrated that the risk of recurrence was significantly higher in patients with >5 LN metastases than in those with <5 LN metastases [24]. Similar findings are reported in several other studies [25, 26]. In light of this concept and the criteria of recurrence risk determined by the ATA [27], we used a cutoff value of at least 6 LNs to select patients for estimation of LNR. Moreover, all the patients included in the LNR analysis were examined with preoperative ultrasound mapping of the cervical LNs to minimize the risk of missing metastases in the lateral cervical compartment. Thus, in contrast to several previous studies based on registry data, the calculation of LNR in this study was estimated in a well-controlled patient cohort with consistent surgical treatment, histopathological assessment, and clinical follow-up.

In selected patient groups, prophylactic central lymph node dissection (CND) increases the risk of postoperative morbidity, such as hypocalcaemia, without evidence of improved locoregional control. Patients who underwent total thyroidectomy without CND exhibited a low risk of locoregional recurrence [28–31]. Moreover, it is known that micrometastases do not affect the clinical outcome of patients with PTC. For example, Viola et al. found high prevalence of micrometastases in clinically apparent node-negative patients, but neither tumor recurrence nor mortality rates were decreased after CND [32]. Interestingly, Chang et al. studied the significance of micrometastasis in the concept of LNR by calculating the LNR with and without including the micrometastases in the number of metastatic LNs. They found that tumor recurrence of PTC could be predicted more accurately if micrometastases were not included in the LNR calculation. Our results are consistent with these findings and show that micrometastases were neither related to advanced tumor stages nor tumor recurrence. Lymph node ratio was significantly higher in patients with ≥T3 tumors compared to those with both T1 and T2 tumors, which is consistent with previous studies [28, 33], supporting the idea that prophylactic CND might not affect the clinical outcome of patients with T1-2 PTC. In light of these observations, we believe that estimation of LNR in patients with T1-2 and LN with micrometastases may have limited clinical benefits but advantageously can be used in dynamic risk estimation for patients with ≥T3 tumors and all patients with preoperatively confirmed pathological LN metastases in whom therapeutic CND is planned.

Another significant finding in this study was that patients who exhibited metastases in lateral cervical LNs only had similar RFS as those with metastasis in central LN (N1a stage). According to the current N stage classification, these patients are considered having N1b stage with increased risk of tumor recurrence [31], which probably is an overestimation of tumor staging. Although this observation is based on a limited number of patients, these results intuitively indicate that the extent of LN metastasis might encompass the malignant behavior of PTC better than the anatomical LN location. To the best of our knowledge, these findings have not been reported elsewhere.

Ki-67 constitutes a prognostic indicator in several types of tumors [9, 34, 35]. In PTC, the Ki-67 index is related to histological variants and clinical manifestations [36]. However, there is no consensus on the optimal cutoff value of Ki-67 associated with prognosis and clinical outcomes of PTC. In this study, a Ki-67 index > 3% was related to the presence of LN metastases and shorter RFS. These findings are consistent with observations reported in previous studies, although the method of detecting and evaluating the Ki-67 index as well as the number of patients included in these studies differ. For example, Ito et al. reported differential association of the Ki-67 index in relation to RFS and disease-free survival, where a Ki-67 index of >1% and >3% were associated with shorter RFS and disease-free survival, respectively [10]. Tang et al. reported in a study of 571 PTC patients that a Ki-67 index ≥ 2.5% was related to shorter RFS and also had diagnostic value when PTC was compared to benign thyroid lesions [11]. Hence, based on findings reported in several PTC studies and experiences from the management of other tumor types, Ki-67 may constitute a useful biological marker in the clinical assessment of PTC.

In several types of solid tumors, accumulating evidence indicates that the biological phenotype of the tumor cells is a superior prognostic factor than the anatomic classification dictated by the TNM stage [37]. High Ki-67 expression in cancer cells is correlated with lymph node metastases [11], but it is unclear whether it is proportional to the number of metastatic LN. In this study, tumors in patients with LNR ≥ 21% exhibited a significantly higher Ki-67 index compared to tumors from patients having LNR < 21%. Moreover, the Ki-67 index was not correlated to N stage. These results suggest that LNR is probably more consistent in predicting the biology of PTC, at least its proliferative activity, compared to N stage.

The current study has several limitations. It lacks histological subtyping pf PTCs, as this was not routinely performed during the study period. The patient cohort was limited with low rates of tumor recurrence, why it was not feasible to perform multivariate analysis to explore the prognostic value of Ki-67 index and LNR. Finally, this is a single-center investigation, and further research is needed to confirm the reproducibility of our findings in other patient cohorts.

## Conclusion

The Ki-67 proliferation index is correlated to LN metastasis and tumor recurrence in PTC. The LNR is associated with tumor recurrence regardless of the anatomical site of cervical LN metastases. These findings indicate that Ki-67 and LNR better reflect the malignant behavior of advanced PTC and constitute useful prognostic indicators in addition to the well-established conventional TNM classification.

## References

[1] Kim M, Jeon MJ, Oh HS, et al (2018) Prognostic implication of N1b Classification in the eighth edition of the tumor-node-metastasis staging system of differentiated thyroid cancer. Thyroid 28:496–503 10.1089/thy.2017.0473 29620964

[2] Hwang HS, Orloff LA (2011). Efficacy of preoperative neck ultrasound in the detection of cervical lymph node metastasis from thyroid cancer. Laryngoscope.

[3] Vas Nunes JH, Clark JR, Gao K (2013). Prognostic implications of lymph node yield and lymph node ratio in papillary thyroid carcinoma. Thyroid.

[4] Aljabery F, Shabo I, Olsson H (2017). Radio-guided sentinel lymph node detection and lymph node mapping in invasive urinary bladder cancer: a prospective clinical study. BJU Int.

[5] Solak M, Turkoz FP, Keskin O (2015). The lymph node ratio as an independent prognostic factor for non-metastatic node-positive breast cancer recurrence and mortality. J BUON.

[6] Roberts TJ, Colevas AD, Hara W (2016). Number of positive nodes is superior to the lymph node ratio and American Joint Committee on Cancer N staging for the prognosis of surgically treated head and neck squamous cell carcinomas. Cancer.

[7] Lee YM, Sung TY, Kim WB (2016). Risk factors for recurrence in patients with papillary thyroid carcinoma undergoing modified radical neck dissection. Br J Surg.

[8] Cardoso F, Senkus E, Costa A (2018). 4th ESO-ESMO International consensus guidelines for Advanced Breast Cancer (ABC 4)dagger. Ann Oncol Off J Eur Soc Med Oncol/ESMO.

[9] Lopez-Aguiar AG, Ethun CG, Postlewait LM (2018). Redefining the Ki-67 Index stratification for low-grade pancreatic neuroendocrine tumors: improving its prognostic value for recurrence of disease. Ann Surg Oncol.

[10] Ito Y, Miyauchi A, Kakudo K (2010). Prognostic significance of ki-67 labeling index in papillary thyroid carcinoma. World J Surg.

[11] Tang J, Gui C, Qiu S (2018). The clinicopathological significance of Ki67 in papillary thyroid carcinoma: a suitable indicator?. World J Surg Oncol.

[12] Taskforce ATAG, on Thyroid N, Differentiated Thyroid C, Cooper DS, (2009). Revised American Thyroid Association management guidelines for patients with thyroid nodules and differentiated thyroid cancer. Thyroid.

[13] Pacini F, Schlumberger M, Dralle H (2006). European consensus for the management of patients with differentiated thyroid carcinoma of the follicular epithelium. Eur J Endocrinol.

[14] Chang GJ, Rodriguez-Bigas MA, Skibber JM (2007). Lymph node evaluation and survival after curative resection of colon cancer: systematic review. J Natl Cancer Inst.

[15] Park YH, Lee SJ, Cho EY (2011). Clinical relevance of TNM staging system according to breast cancer subtypes. Ann Oncol Off J Eur Soc Med Oncol/ESMO.

[16] Shang X, Liu J, Li Z (2019). A hypothesized TNM staging system based on the number and location of positive lymph nodes may better reflect the prognosis for patients with NSCLC. BMC Cancer.

[17] Schneider DF, Mazeh H, Chen H (2013). Lymph node ratio predicts recurrence in papillary thyroid cancer. Oncologist.

[18] de Leede EM, Sibinga Mulder BG, Bastiaannet E (2016). Common variables in European pancreatic cancer registries: The introduction of the EURECCA pancreatic cancer project. Eur J Surg Oncol.

[19] Rubinstein JC, Dinauer C, Herrick-Reynolds K (2019). Lymph node ratio predicts recurrence in pediatric papillary thyroid cancer. J Pediatr Surg.

[20] Ryu IS, Song CI, Choi SH (2014). Lymph node ratio of the central compartment is a significant predictor for locoregional recurrence after prophylactic central neck dissection in patients with thyroid papillary carcinoma. Ann Surg Oncol.

[21] Vinh-Hung V, Verschraegen C, Promish DI (2004). Ratios of involved nodes in early breast cancer. Breast Cancer Res.

[22] Adam MA, Pura J, Goffredo P (2015). Presence and number of lymph node metastases are associated with compromised survival for patients younger than age 45 years with papillary thyroid cancer. J Clin Oncol.

[23] Feinstein AR, Sosin DA, Wells CK (1984). The Will Rogers phenomenon: improved technologic diagnosis and stage migration as a source of nontherapeutic improvement in cancer prognosis. Trans Assoc Am Physicians.

[24] Sugitani I, Kasai N, Fujimoto Y (2004). A novel classification system for patients with PTC: addition of the new variables of large (3 cm or greater) nodal metastases and reclassification during the follow-up period. Surgery.

[25] Leboulleux S, Rubino C, Baudin E (2005). Prognostic factors for persistent or recurrent disease of papillary thyroid carcinoma with neck lymph node metastases and/or tumor extension beyond the thyroid capsule at initial diagnosis. J Clin Endocrinol Metab.

[26] Ito Y, Fukushima M, Tomoda C (2009). Prognosis of patients with papillary thyroid carcinoma having clinically apparent metastasis to the lateral compartment. Endocr J.

[27] Haugen BR, Alexander EK, Bible KC (2016). 2015 American Thyroid Association Management guidelines for adult patients with thyroid nodules and differentiated thyroid cancer: the American Thyroid Association guidelines task force on thyroid nodules and differentiated thyroid cancer. Thyroid.

[28] Conzo G, Tartaglia E, Avenia N (2016). Role of prophylactic central compartment lymph node dissection in clinically N0 differentiated thyroid cancer patients: analysis of risk factors and review of modern trends. World J Surg Oncol.

[29] Vrachimis A, Wenning C, Gerss J (2015). Not all DTC patients with N positive disease deserve the attribution "high risk". Contribution of the MSDS trial. J Surg Oncol.

[30] Nixon IJ, Wang LY, Palmer FL (2014). The impact of nodal status on outcome in older patients with papillary thyroid cancer. Surgery.

[31] Smith VA, Sessions RB, Lentsch EJ (2012). Cervical lymph node metastasis and papillary thyroid carcinoma: does the compartment involved affect survival? Experience from the SEER database. J Surg Oncol.

[32] Viola D, Materazzi G, Valerio L (2015). Prophylactic central compartment lymph node dissection in papillary thyroid carcinoma: clinical implications derived from the first prospective randomized controlled single institution study. J Clin Endocrinol Metab.

[33] Sywak M, Cornford L, Roach P et al (2006) Routine ipsilateral level VI lymphadenectomy reduces postoperative thyroglobulin levels in papillary thyroid cancer. Surgery 140:1000–1005. (Discussion 1005–1007) 10.1016/j.surg.2006.08.001 17188149

[34] Luporsi E, André F, Spyratos F (2012). Ki-67: level of evidence and methodological considerations for its role in the clinical management of breast cancer: analytical and critical review. Breast Cancer Res Treat.

[35] Berney DM, Gopalan A, Kudahetti S (2009). Ki-67 and outcome in clinically localised prostate cancer: analysis of conservatively treated prostate cancer patients from the Trans-Atlantic Prostate Group study. Br J Cancer.

[36] Müssig K, Wehrmann T, Dittmann H (2012). Expression of the proliferation marker Ki-67 associates with tumour staging and clinical outcome in differentiated thyroid carcinomas. Clin Endocrinol (Oxf).

[37] Mittendorf EA, Ballman KV, McCall LM (2015). Evaluation of the stage IB designation of the American Joint Committee on cancer staging system in breast cancer. J Clin Oncol.

